# 3D-printed tissue-simulating phantoms for near-infrared fluorescence imaging of rheumatoid diseases

**DOI:** 10.1117/1.JBO.27.7.074702

**Published:** 2022-06-16

**Authors:** Sandra Schädel-Ebner, Ole Hirsch, Thomas Gladytz, Dirk Gutkelch, Kai Licha, Jörn Berger, Dirk Grosenick

**Affiliations:** a Physikalisch-Technische Bundesanstalt (PTB), Berlin, Germany; b Hochschule für angewandte Wissenschaft und Kunst Hildesheim/Holzminden/Göttingen (HAWK), Fakultät Ingenieurwissenschaften und Gesundheit, Göttingen, Germany; c Max-Delbrück-Centrum für Molekulare Medizin in der Helmholtz-Gemeinschaft (MDC), Berlin, Germany; d FEW Chemicals GmbH, Bitterfeld-Wolfen, Germany; e Xiralite GmbH, Berlin, Germany

**Keywords:** tissue phantoms, three-dimensional printing, fluorescence imaging, absorption, scattering

## Abstract

**Significance:**

Fluorescence imaging of rheumatoid diseases with indocyanine green (ICG) is an emerging technique with unique potential for diagnosis and therapy. Device characterization, monitoring of the performance, and further developments of the technique require tissue-equivalent fluorescent phantoms of high stability with appropriate anatomical shapes.

**Aim:**

Our investigations aim at the development of a three-dimensional (3D) printing technique to fabricate hand and finger models with appropriate optical properties in the near-infrared spectral range. These phantoms should have fluorescence properties similar to ICG, and excellent photostability and durability over years.

**Approach:**

We modified a 3D printing methacrylate photopolymer by adding the fluorescent dye Lumogen IR 765 to the raw material. Reduced scattering and absorption coefficients were adjusted to values representative of the human hand by incorporating titanium dioxide powder and black ink. The properties of printed phantoms of various compositions were characterized using UV/Vis and fluorescence spectroscopy, and time-resolved measurements. Photostability and bleaching were investigated with a hand imager. For comparison, several phantoms with ICG as fluorescent dye were printed and characterized as well.

**Results:**

The spectral properties of Lumogen IR 765 are very similar to those of ICG. By optimizing the concentrations of Lumogen, titanium dioxide, and ink, anatomically shaped hand and vessel models with properties equivalent to *in vivo* investigations with a fluorescence hand imager could be printed. Phantoms with Lumogen IR 765 had an excellent photostability over up to 4 years. In contrast, phantoms printed with ICG showed significant bleaching and degradation of fluorescence over time.

**Conclusions:**

3D printing of phantoms with Lumogen IR 765 is a promising method for fabricating anatomically shaped fluorescent tissue models of excellent stability with spectral properties similar to ICG. The phantoms are well-suited to monitor the performance of hand imagers. Concepts can easily be transferred to other fluorescence imaging applications of ICG.

## 1 Introduction

Fluorescence imaging has a long history in diffuse optics.^1^^–^^4^ The main success of this technology in clinical applications is based on the broad acceptance of the contrast agent indocyanine green.^5^^,^^6^ This fluorescent dye is applied as a marker for cancer tissue^7^^–^^10^ and for sentinel lymph node detection in fluorescence-guided surgery,^11^^–^^13^ and as a perfusion agent, e.g., for the detection of rheuma.^14^^,^^15^ With its absorption and fluorescence band in the near-infrared (NIR) spectral region the application of indocyanine green (ICG) is not limited to superficial tissue regions. It can be also used for imaging through even centimeter thick tissue regions as successfully demonstrated by *in vivo* breast tumor characterization.^5^^,^^16^

These advantages make ICG to a well-suited agent for the detection of rheumatoid diseases from perfusion measurements. In this application, fluorescence images of both hands of the subject are recorded over time to determine the flow pattern of an ICG bolus through the blood vessels of the fingers. The patterns are used then to get information on the inflammation of the finger joints. In the past, we developed two types of fluorescence imagers, a simple wide-field system with camera detection, and a scanning system with point-like illumination and fluorescence detection.^14^^,^^17^^,^^18^ Over the years, the technique is coming into use by more and more rheumatologists.^15^

Similar to the general need for phantoms for fluorescence-guided surgery,^19^^–^^21^ fluorescent phantoms are also of high interest for rheumatism imaging. Phantoms with stable properties over years available at the location of a device would allow for regular monitoring of the device performance and would be very helpful in case of malfunctions, with side effects on service costs. Besides, realistic hand-shaped phantoms are required to investigate and correct issues related to the cylindrical shape of the fingers which hampers a homogeneous illumination of the tissue. Furthermore, phantoms with vessel-like fluorescent structures enable to study of the depth sensitivity of the imager to correctly interpret fluorescence from vessels located at different depths. The requirements for the special shapes make such phantoms a candidate for three-dimensional (3D) printing.^22^^–^^26^

Generally, the effect of light scattering and the intrinsic tissue absorption, e.g., by oxy- and deoxyhemoglobin, confound the linearity between dye concentration and detected fluorescent light. Therefore, the phantoms should have scattering and absorption properties in the ∼760 to 830 nm spectral range close to those of finger and hand tissue. The requirement of adjusting the scattering and absorption properties to the tissue of interest has been solved in 3D printing of phantoms for several applications by adding suited materials to the original printing materials.^27^^,^^28^

A more challenging task is the realization of stable fluorescence properties in 3D printing comparable to the fluorophore ICG since ICG itself is known to be of low stability in various environments. The inclusion of hollow tubes in 3D-printed nonfluorescent materials to be filled with ICG solutions is a practicable way for short-term laboratory studies but less suited for long-term stability.^29^^–^^34^

Important progress was achieved in the design of stable phantoms for NIR fluorescence-guided surgery conventionally fabricated with polyurethane as base material by using fluorescent quantum dots.^20^ A main disadvantage of this approach is the considerable deviation of the absorption spectrum of the fluorescent substance from that of ICG. Recently, ICG (IR-125 laser dye from Exciton Inc., Lockburne, Ohio, United States) was successfully applied as fluorophore for both conventional solid phantoms based on polyurethane,^35^ and 3D-printed solid phantoms based on methacrylate resins.^36^ During preparation the fluorophore was dissolved in dimethyl sulfoxide (DMSO) which is known to yield stable ICG solutions for laser emission.^37^^–^^39^ For the polyurethane phantoms an average decrease of fluorescence intensity of about 5% within 2 months was reported, and no more change after the next 3 months. The 3D-printed phantoms (resin with ICG) showed random deviations of ±4%.

In all, the reported stability of ICG-style solid phantoms in the order of 5% is promising but not yet sufficient for device monitoring over months to years. To get long-time stability in 3D-printed fluorescence phantoms with spectral properties close to those of ICG better than 1%, we apply the dye Lumogen IR 765, which was originally developed for laser cutting of plastics. Phantoms with optical properties being similar to those of finger and hand tissue in the NIR spectral range are obtained from a commercial methacrylate photopolymer by adding this dye together with scattering microparticles [titanium dioxide (${\mathrm{TiO}}_{2}$) powder] and an absorbing dye (black ink) to the raw material prior to the printing process. We demonstrate the method by fabricating 3D-printed phantoms of various shapes with high repeatability. The fluorescence properties and the optical properties in the NIR are characterized by spectroscopic measurements and by the time-resolved method, respectively. Based on our first exploratory investigations started several years ago,^40^ we discuss the long-term stability of the phantoms over a period of four years. We also compare photostability and bleaching properties to phantoms printed with ICG as a fluorescent dye.

## 2 Materials and Methods

A commercial digital light processing (DLP) 3D printer (Perfactory^®^ 4 DSP XL, EnvisionTEC GmbH, Germany) that uses a clear UV-cure methacrylate photopolymer resin including a photoinitiator (E-Shell 600, EnvisionTEC GmbH, Germany) as base material was utilized to fabricate phantoms for this study. Titanium dioxide (${\mathrm{TiO}}_{2}$) powder (Merck KGaA, Germany) and black India ink (Higgins Black India, Chartpak, United States) were applied as scattering and absorption agents, respectively. Fluorescence properties were adjusted by adding the dye Lumogen IR 765 (BASF SE, Germany).

Lumogen IR 765 is a highly transparent NIR selective absorber dye with high absorption efficiencies and very little residual color in the visible range. It is a member of the group of rylene dyes, which are utilized because of their intense absorption, high photostability, and high fluorescence quantum yield.^41^ Due to these properties they have been used as industrial pigments for many years.^42^ Lumogen IR 765 is a well-established member of the BASF Lumogen^®^IR product line, a novel generation of additives especially designed for applications where high NIR absorption is required, such as laser welding of plastics.^43^ It is compatible with most standard polymers, and, as will be shown as follows, has optical characteristics similar to ICG,^44^ which is frequently used in fluorescence-assisted diagnostics and therapy. The advantage of Lumogen is its excellent UV and heat stability even in polymer applications, providing the opportunity of the addition to 3D printing base materials for a feasible fabrication of solid fluorescent phantoms with different geometries. Additionally, it is extremely resistant to external chemical and physical influences. Lumogen IR 765 is free of halogens and heavy metals and toxicologically harmless, making it suitable for the medical device technology.

The concentrations of ${\mathrm{TiO}}_{2}$, ink, and Lumogen needed to realize phantom properties being similar to the conditions of ICG perfusion measurements on human fingers were estimated by sequentially printing and analyzing regular-shaped phantoms with selected mixing ratios of the ingredients. The desired optical parameters for human fingers around 800 nm are $\mu_{a}\approx 0.06\ldots 0.1\;{\mathrm{cm}}^{-1}$ and $\mu_{s}^{\prime }\approx 9\ldots 10\;{\mathrm{cm}}^{-1}$. For each print job, carefully weighted amounts of the ingredients were successively added to a known mass of resin. Prior to use, Lumogen was dissolved into a small amount of trichlormethane (${\mathrm{CHCl}}_{3}$) to enhance the dispersion process. After manually mixing and using a magnetic stirrer, the resin suspension was placed in an ultrasonic bath for 3 to 4 h. To prevent the ${\mathrm{TiO}}_{2}$ and ink pigments from settling, the mixture was additionally stirred thoroughly by hand several times during this period. As we could elicit in preliminary experiments, despite stirring, vacuum degassing was not required to remove air bubbles. Subsequently, the mixture was transferred into the resin tank of the printer, where a UV laser cures it at the desired positions forming each layer of the 3D print (curing intensity of $190\;{\mathrm{mW}/\mathrm{cm}}^{2}$).

**Fig. 1:**
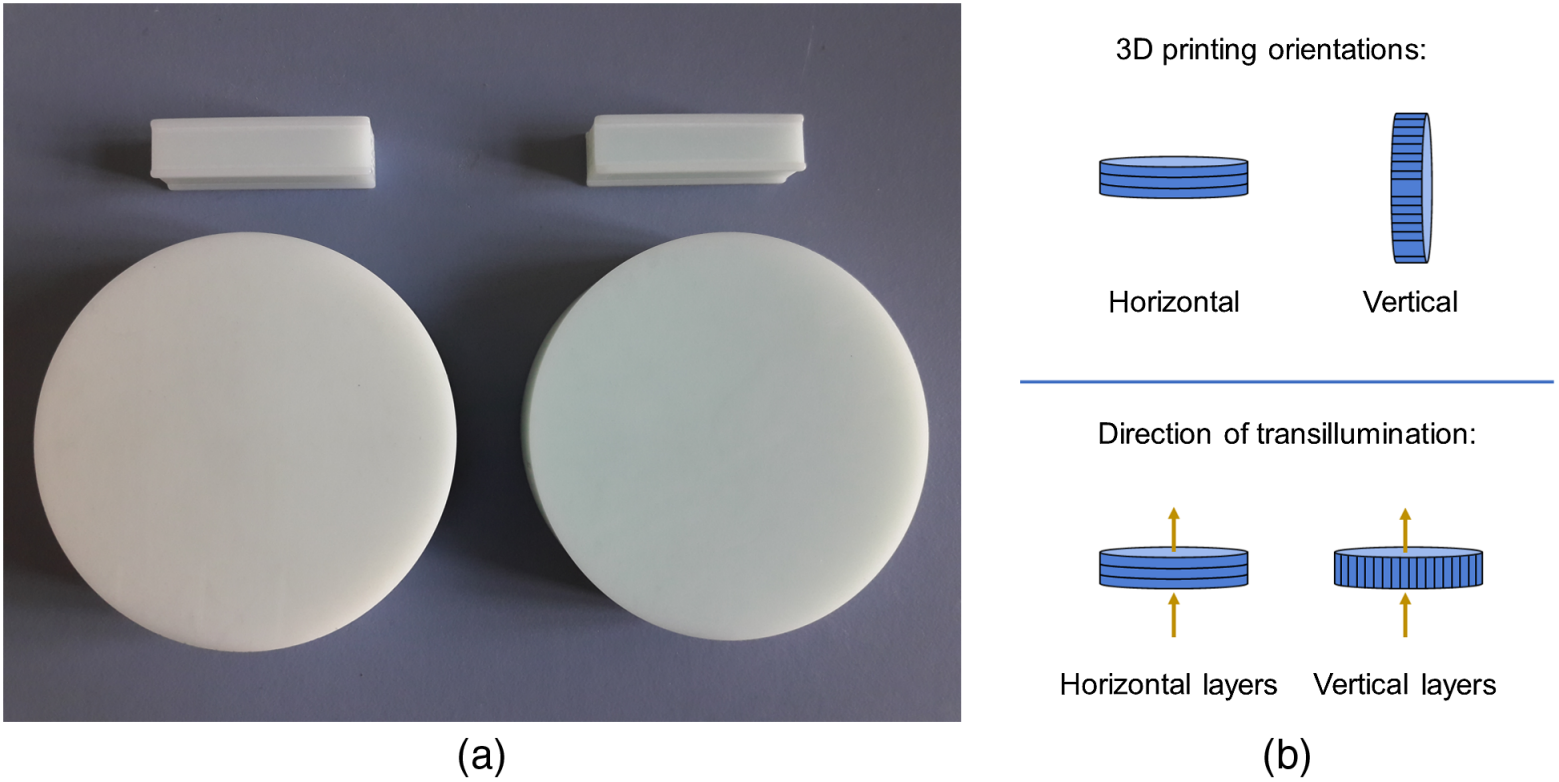
(a) Printed disc and cuvette-shaped phantoms made from a mixture of clear UV-cure methacrylate photopolymer resin, Titanium dioxide (${\mathrm{TiO}}_{2}$) powder, black India ink, and Lumogen dye. Left: 2  mg/g ${\mathrm{TiO}}_{2}+0.5\;\mu L/g\text{ ink}+1.5\;\mu g/g$ Lumogen. Right: $2\;\mathrm{mg}/g{\mathrm{TiO}}_{2}+0.5\;\mu L/g\text{ ink}+5\;\mu g/g$ Lumogen. (b) Illustration of the internal layer orientation for the two printing configurations of the discs (top) and of the light propagation direction in the optical characterization measurements (bottom).

For each mixing ratio solid cylindrical phantoms (discs) with a thickness of 2 cm and a diameter of 8 cm [Fig. 1(a)] as well as cuvette-shaped phantoms (cuboids with a 10 mm squared cross-section) were printed for systematic investigations of the phantom properties. Each time two printing orientations were used: horizontal and vertical with respect to the printing bed [Fig. 1(b)]. After each printing process, a postcuring of the phantoms in a UV lightbox (Otoflash G171, Dentona, Germany) was done with an intensity of about $28\;{\mathrm{mW}/\mathrm{cm}}^{2}$, as recommended in the printing instructions. This UV light treatment improves complete polymer conversion by reducing the residual monomer to a minimum and ensures the final material properties such as high durability. Afterward, the phantom was milled and polished to a roughness of 0.1  μm to enhance surface quality.

Fluorescence spectra of mixtures from Lumogen in liquid resin and ${\mathrm{CHCl}}_{3}$, respectively, were measured with cuvettes (10  mm×10  mm×45  mm) by a fluorometer (Fluorolog, Horiba) between 750 and 900 nm using an excitation wavelength of 740 nm. The values of the solid fluorescent phantoms (printed in the form of cuvettes) were obtained by performing fluorescence measurements using front face detection. Absorbance spectra of the phantoms printed without ${\mathrm{TiO}}_{2}$ powder and ink were recorded by a UV/VIS spectrophotometer (Cary, Agilent) between 550 and 900 nm.

Time-resolved measurements of diffuse transmittance were applied to determine the reduced scattering coefficient $\mu_{s}^{\prime }$ and the absorption coefficient $\mu_{a}$ of the printed disc phantoms containing ${\mathrm{TiO}}_{2}$ powder and ink as additives. Distributions of times of flight of photons (DTOFs) were recorded at the center position of the phantoms at selected wavelengths in the NIR spectral range (710, 750, 800, 850, and 900 nm) using a femtosecond Ti:Sapphire laser and time-correlated single-photon counting with an overall time resolution of 35 ps. The measured DTOFs were analyzed by using a Monte Carlo model of light propagation. To this end, a lookup table (LUT) of simulated DTOFs was created with $\mu_{s}^{\prime }$ values ranging from 0.1 to $20\;{\mathrm{cm}}^{-1}$ in steps of $0.1\;{\mathrm{cm}}^{-1}$ while $\mu_{a}$ was set to zero (white Monte Carlo). Absorption was included during the fit by multiplying the white Monte Carlo DTOFs with the time dependent factor $\exp(-\mu_{a}c_{M}t)$ with $c_{M}$ denoting the speed of light in the phantom. To assess the homogeneity of the phantoms DTOFs were additionally recorded in transmission along a two-dimensional scan grid with a step size of 5 mm.

A homemade prototype for fluorescence imaging of human hands was used to compare the detector fluorescence counts obtained from the cuvette-like phantoms with different Lumogen concentrations with fluorescence measurements on humans acquired in previous clinical studies with ICG as a tracer.^17^^,^^18^ Briefly, this device has a covered dark space to put in both hands of the patient. The backs of the flat hands and fingers are illuminated by two groups of air-cooled LEDs with an emission wavelength centered at 740 nm. A scientific camera equipped with a long-pass filter records images of the fluorescence emission. Exposure time, camera gain, and image repetition rate can be varied with high flexibility.

The hand imager was also used for investigations on photostability of the printed phantoms. To this end, fluorescence from selected cuvette-shaped phantoms was regularly imaged during the first month after fabrication. Some of the cuvettes were stored in a dark environment; others under daylight conditions. For selected phantoms, long-term stability was checked over a period of up to 4 years. Besides, fluorescence bleaching was investigated by quasicontinuous excitation over one hour. To evaluate the advantages and disadvantages of Lumogen based phantoms with respect to ICG, we also printed cuvette-shaped phantoms with ICG as fluorescent dye using concentrations of 20 and 100  nmol/L. Before mixing ICG (Thermo Scientific IR-125, ACROS Organics) with the resin the dye was dissolved in DMSO. The ${\mathrm{TiO}}_{2}$ and ink concentration were set to 2  mg/g and 0.5  μL/g, respectively. Printing and UV curing was done in the same way as for the Lumogen phantoms. The ICG phantoms were used in parallel to Lumogen phantoms in photostability measurements. For each fluorescence image regions of interest were defined containing the different phantoms, and fluorescence intensities were calculated as mean of the 21 pixels with the highest brightness.

Based on the various investigations on the disc- and cuvette-shaped phantoms, concentrations of 1.5  μg Lumogen/g resin, 2 mg ${\mathrm{TiO}}_{2}/g$ resin, and 0.5  μL  ink/g resin were selected to be appropriate for printing phantoms with shapes relevant for performance characterization, optimization, and inspection of hand imagers as well as for related basic investigations. To print models of the left and right hands the outer shape of both hands of a volunteer was scanned with a commercial 3D surface scanner (Artec Eva, Artec 3D, Luxembourg, Europe). The 3D data were converted to the data format required for the printer. Furthermore, a 3D data set of the blood vessels in a human finger (Shutterstock, Inc., New York, United States) was used to print a representative vessel structure. Finally, cylinders of 8-cm length with diameters of 1, 2, and 3 mm were printed with holders at both ends suited for basic investigations such as depth sensitivity when put into a scattering and absorbing liquid or when placed inside a solid finger-like phantom made, e.g., from a mixture of PDMS, ${\mathrm{TiO}}_{2}$ and ink by the help of a mold. In each print job also cuvette-shaped phantoms were made in parallel to monitor the properties of the realistic shaped objects.

## 3 Results and Discussion

Main requirements on phantoms for monitoring the performance of ICG-based fluorescence hand imagers as well as for the comparison of different devices are (i) a fluorescence and absorbance spectral characteristic similar to ICG, (ii) background absorption and scattering properties comparable to the human hand, (iii) long term stability of the properties including photostability, and (iv) a sufficient homogeneity and reproducibility. Therefore, we first focus on the spectral properties of the regularly shaped phantoms, followed by results on photostability, bleaching, and homogeneity. At the end, we present the examples of realistic phantoms with fluorescence, absorption, and scattering properties similar to the human hand.

### 3.1 Fluorescence Spectra of Lumogen in Liquid Resin and Chloroform

**Fig. 2:**
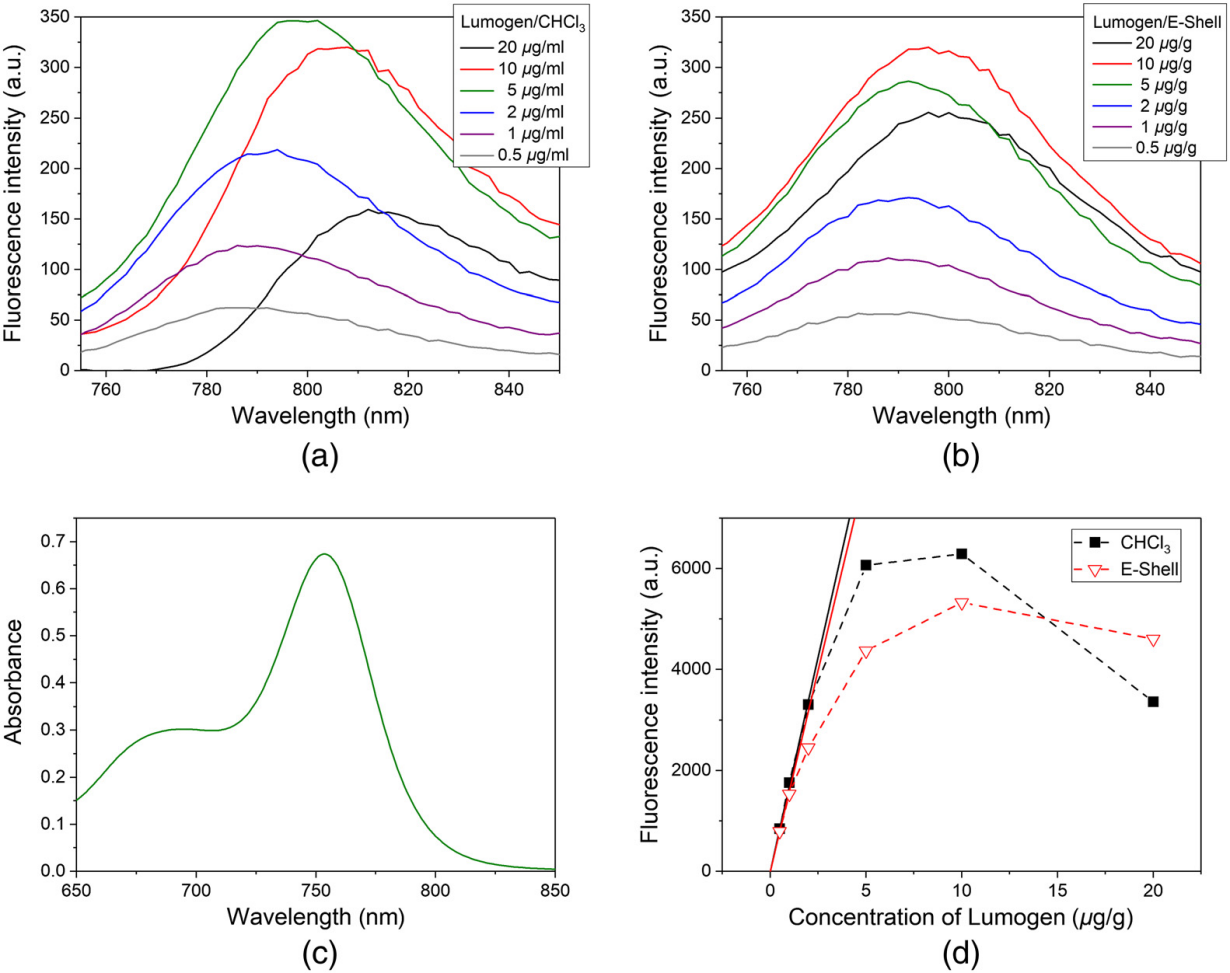
Dependence of fluorescence intensity on the concentration of Lumogen in (a) chloroform and (b) uncured E-Shell at an excitation wavelength of $\lambda_{\mathrm{Ex}}=740\;\mathrm{nm}$. (c) Absorption spectrum of Lumogen in chloroform (5  μg/mL). (d) Concentration-dependent saturation effect (fluorescence intensity for λ>800  nm). Solid lines illustrate linearity at small concentrations.

Fluorescence spectra of Lumogen dispersed in ${\mathrm{CHCl}}_{3}$ and in uncured E-Shell are given in Figs. 2(a) and 2(b). The maximum emission lies at about 790 nm at a low concentration. The spectral half-width amounts to about 60 nm. With increasing concentration, the peak emission shifts slightly toward 798 nm. Figure 2(c) shows the absorption spectrum of Lumogen in ${\mathrm{CHCl}}_{3}$ having its maximum at 754 nm. The large overlapping of the absorption and fluorescence spectra leads to a remarkable reabsorption of the emitted fluorescence by Lumogen itself accompanied by a concentration quenching with a saturation effect (starting above 2  μg/mL in ${\mathrm{CHCl}}_{3}$ and 2  μg/g in E-Shell, respectively) and a bathochromic shift of the spectra. The effect of saturation is illustrated in Fig. 2(d) in which the fluorescence intensity is plotted versus dye concentration assuming long-pass filtering (λ>800  nm). Generally, saturation is stronger for Lumogen dissolved in ${\mathrm{CHCl}}_{3}$. Deviations from linearity between fluorescence intensity and concentration start at about 2.5  μg/mL for ${\mathrm{CHCl}}_{3}$ and 1.5  μg/g for E-Shell.

### 3.2 Absorbance and Fluorescence Spectra of Printed Phantoms

**Fig. 3:**
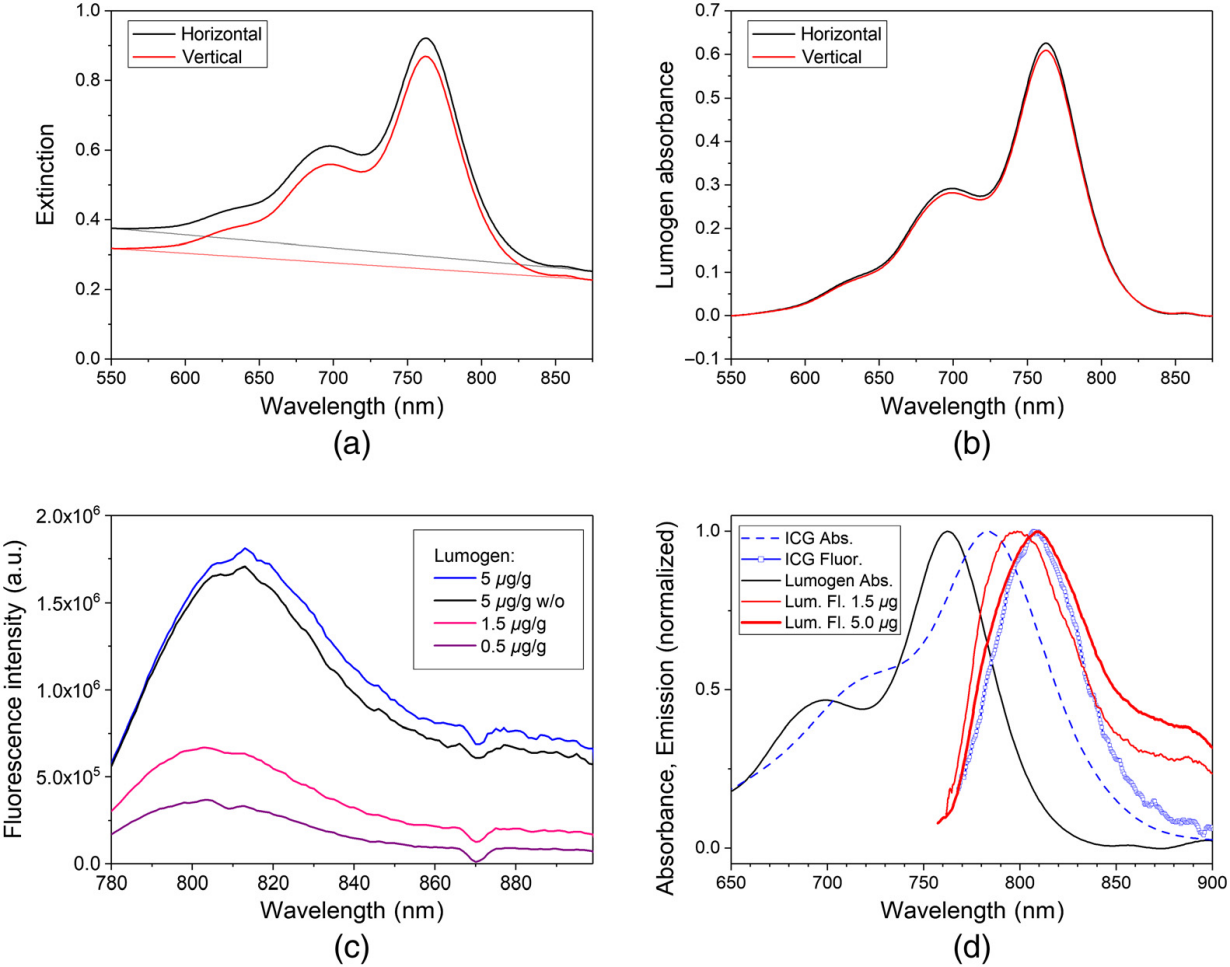
(a) Extinction spectra of printed cuvette-shaped phantoms with 5  μg/g Lumogen in E-Shell 600 (without additives) in horizontal (black) and vertical (red) fabrication direction during the printing process. (b) Absorbance spectra after background correction. (c) Fluorescence spectra of printed phantoms containing $2\;\mathrm{mg}/g\;{\mathrm{TiO}}_{2}+0.5\;\mu L/g$ ink in E-Shell 600 with different concentrations of Lumogen. Black line: reference sample with 5  μg/g Lumogen in E-Shell 600 without additives. $\lambda_{\mathrm{Ex}}=740\;\mathrm{nm}$. (d) Comparison of normalized absorbance and fluorescence spectra of printed Lumogen phantoms with spectra of ICG (2  μmol/L) in water. Absorbance: 5  μg/g Lumogen in E-Shell 600, without additives; Fluorescence: 1.5  μg/g (thin red line) and 5  μg/g (thick red line) Lumogen in E-Shell 600, with additives.

In Fig. 3(a), extinction spectra of printed cuboid phantoms made from E-Shell and Lumogen (without the addition of ${\mathrm{TiO}}_{2}$ and ink) are displayed for the horizontal and vertical printing directions. The spectra show a strong background arising essentially from light scatter in the E-Shell material itself. This background can approximately be described by the straight lines in Fig. 3(a). The background is stronger for the horizontal print direction since the internal layer structure of the base material is perpendicularly oriented to the direction of transillumination for this case. This layered substructure results in remarkable light scatter. The effect of the layers was also seen in experiments on cuboids printed from the clear resin without any additives. Transmission measurements with a small diameter laser beam (HeNe laser, 633 nm) being parallel to the internal layer surfaces show a grating-like classical diffraction pattern. The diffraction angles of the maxima were in good agreement with the values expected from the layer thickness used in the printing process. The absorbance contribution of Lumogen shown in Fig. 3(b) is obtained after background subtraction. As expected, there is no essential difference between both print directions. The absorbance spectra of the cured phantoms show a slight shift of the peak absorption as well of the short-wavelength shoulder by 8 nm to the right compared with the spectrum of Lumogen dissolved in ${\mathrm{CHCl}}_{3}$ [see Fig. 2(c)]. The absorption peak is now at 762 nm.

In Fig. 3(c), fluorescence spectra of printed disc phantoms are displayed for several Lumogen concentrations. Apart from the control sample given in black the disc phantoms contain also ${\mathrm{TiO}}_{2}$ powder and ink to realize scattering and absorption properties similar to tissue. In contrast to the uncured resin containing Lumogen [see Fig. 2(b)] with an emission maximum at 798 nm, the maximum of cured resin with Lumogen is shifted to about 805 nm at the lower concentrations. For a Lumogen concentration of 5  μg/g, the peak emission occurs around 814 nm most probably due to the onset of fluorescence reabsorption [saturation effect as discussed in Fig. 2(d)].

Figure 3(d) illustrates the similarity of the absorption and fluorescence properties of the printed Lumogen phantoms to those of ICG. The absorption maximum of Lumogen IR 765 is at 764 nm compared with 784 nm for ICG. Both dyes can be effectively excited with wavelengths in the 700- to 780-nm range. The fluorescence maxima appear nearly at the same wavelength. Hence, Lumogen is well suited for 3D printing of phantoms for tests and applications related to the clinical use of ICG. The fluorescence spectrum for 0.5  μg/g Lumogen (not shown) fits well to that for 1.5  μg/g. The spectral shift at 5  μg/g to longer wavelengths indicates that at this concentration linearity is no longer present due to reabsorption of fluorescence. At 750-nm excitation the fluorescence emission of the 1.5  μg/g Lumogen phantom is expected to be similar to an ICG concentration of 85  nmol/L.

### 3.3 Scattering and Absorption Coefficients of Printed Phantoms

**Fig. 4:**
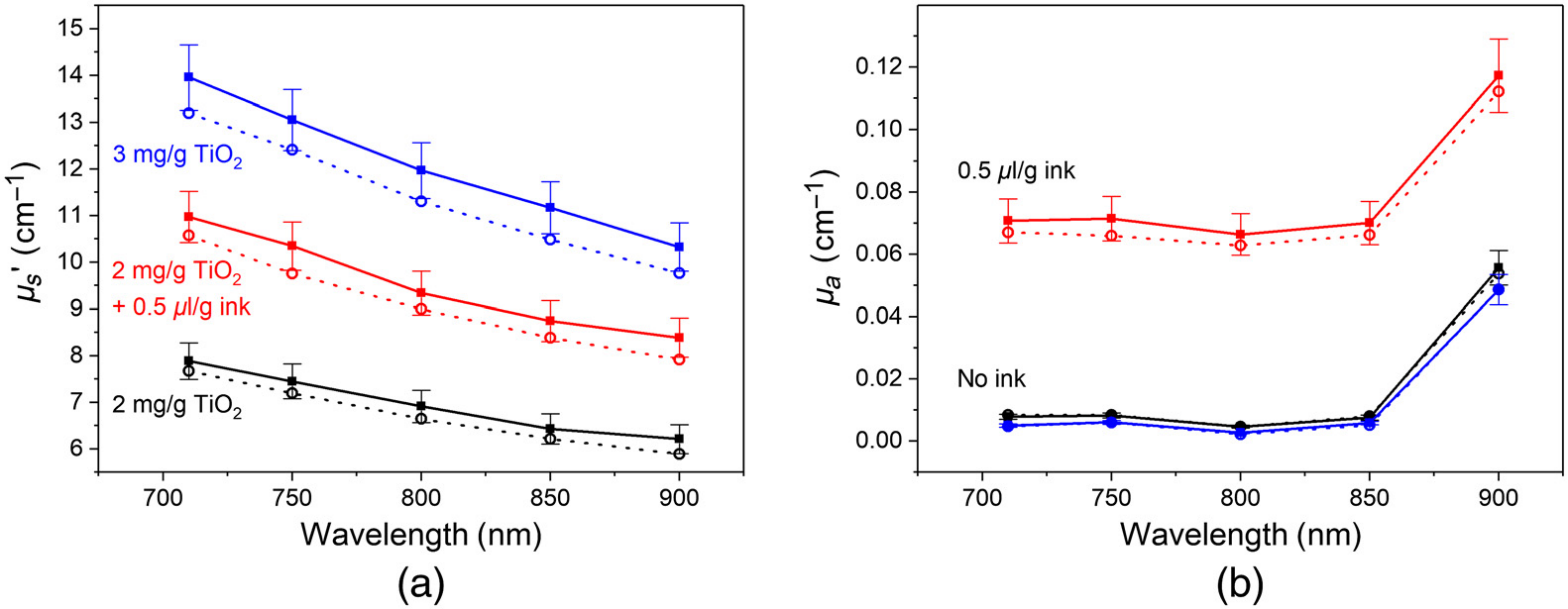
Results for the wavelength-dependent scattering (a) and absorption (b) coefficients of printed disc phantoms at different concentrations of ${\mathrm{TiO}}_{2}$ and ink: 2  mg/g ${\mathrm{TiO}}_{2}$ (black), $2\;\mathrm{mg}/g{\mathrm{TiO}}_{2}+0.5\;\mu L/g$ ink (red), 3  mg/g ${\mathrm{TiO}}_{2}$ (blue) in E-Shell. Filled and open squares represent the horizontal and vertical fabrication direction of the phantom during the printing process. Error bars for vertical print direction (not shown for clarity) have about the same size as for horizontal print direction.

Figure 4 shows the results of the reduced scattering and absorption coefficients of printed disc phantoms including different ${\mathrm{TiO}}_{2}$ and ink concentrations in the 700- to 900-nm spectral range. These investigations were done on phantoms without Lumogen. According to Fig. 4(a), the reduced scattering coefficient decreases with increasing wavelength and increases with the ${\mathrm{TiO}}_{2}$ concentration. The addition of black ink provided, besides the expected absorption, an additional contribution to the scattering coefficient (red curve). Its remarkable increase cannot be explained by the action of ink particles as additional light scatterers. As mentioned by Di Ninni et al.,^45^ scattering of India ink is negligible with respect to absorption and there is only a marginal effect of dependent scattering on the optical properties in liquid tissue phantoms. More likely, the ink influences the propagation of the UV light through the phantom during printing and changes the curing process. It can be assumed that the polymerization of the methacrylate is affected by these changes, which causes the observed increase of light scattering. Also, the refractive index of the resin mixture could be affected having an impact on the scattering properties.

Figure 4(b) illustrates that the absorption can easily be adjusted by the addition of ink. The base absorption without ink is below $0.01\;{\mathrm{cm}}^{-1}$. Beyond 850 nm a major increase of the absorption coefficient occurs due to the base absorption of the photopolymer raw material. Samples were printed in two orientations: horizontal and vertical directions with respect to the printing bed, illustrated as filled and open squares, respectively. Horizontally oriented phantoms show slightly increased scattering and absorption coefficients due to the 3D printing process, which deposits the material layer by layer. Therefore, the light needs to pass through the interfaces between the multiple photopolymer layers of the printing process differently, depending on the orientation. This contribution of the oriented layers was already seen for the polymer base material as discussed in Figs. 3(a) and 3(b) above. Additionally, the longer printing time for the vertical print direction could result in smaller concentrations of the additives due to settling.

### 3.4 Photostability and Bleaching

**Fig. 5:**
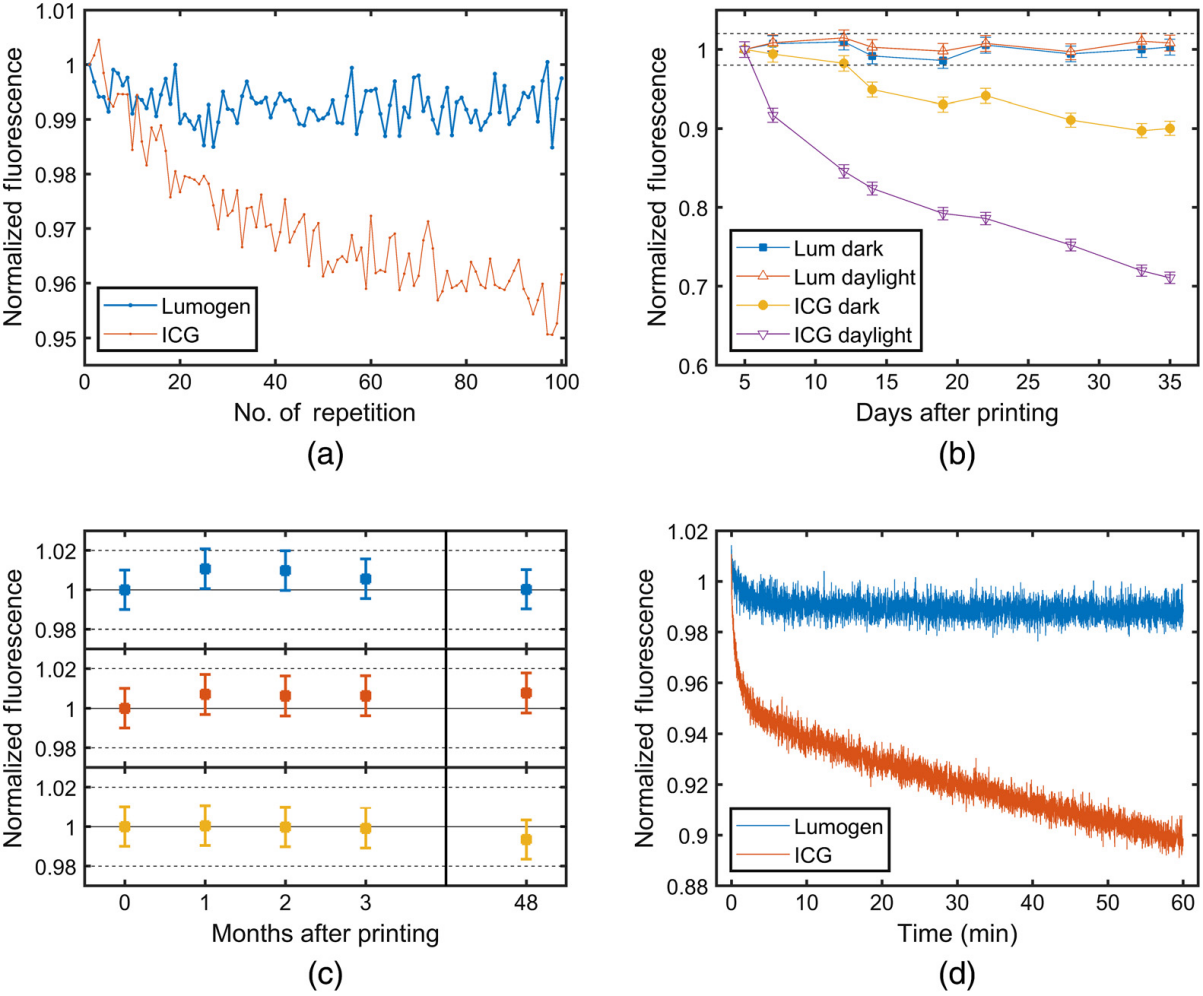
Photostability and bleaching studies on cuvette-shaped phantoms carried out with the hand imager. (a) Normalized fluorescence for a measurement with 100 repetitions (Lumogen 1.5  μg/g and ICG 100  nmol/L); (b) Day-to-day stability of fluorescence for Lumogen (1.5  μg/g) and ICG (100  nmol/L) with the phantoms stored either in the dark or under daylight; (c) Long term-stability of Lumogen phantoms (from top to bottom: Lumogen concentration 5  μg/g, 1.5  μg/g, and 0.5  μg/g); (d) Bleaching behavior of Lumogen (1.5  μg/g) and ICG (100  nmol/L) phantoms over 60 min of pulsed exposure.

The accuracy of the photostability measurements is limited by the stability and noise of the hand imager itself, which depend mainly on the characteristics of the high-power LEDs and the CCD camera. Figure 5(a) illustrates the overall noise, i.e., the combined contribution from the device and the phantom for a Lumogen phantom (dye concentration 1.5  μg/g) and an ICG phantom (dye concentration 100  nmol/L) placed together in the imager. A total of 100 fluorescence images were recorded with an exposure time of 200 ms followed by a pause of about 600 ms to avoid heating of the LEDs. Fluorescence counts were normalized to the value of the first measurement. The Lumogen signal in Fig. 5(a) appears to be constant over time, the standard deviation is 0.35%. Perhaps, a small decrease with time by about 0.5% could be seen, most likely due to LED power drift by marginal warming during emission. From these data, the overall noise of a single measurement with the hand imager was estimated to be about 1%. Fluorescence of the ICG phantom drops down within the short sequence by about 4.5% indicating instable behavior of this phantom.

Figure 5(b) illustrates the stability of Lumogen and ICG phantoms over a period of about 30 days. Each data point is marked with the ±1% uncertainty estimated above. In case of Lumogen fluorescence intensity is always within the ±2% interval indicated by the dashed lines. This result is representative of both the phantom stored in the dark between measurements and the phantom stored under daylight. Fluorescence intensity of the ICG phantom stored in the dark drops down by about 10% within 1 month. The intensity decrease reaches even 30% when the phantom is stored under daylight conditions.

Selected phantoms with Lumogen were also investigated over longer times. The results in Fig. 5(c) indicate that fluorescence emission from Lumogen is even stable after 48 months. The three phantoms in Fig. 5(c) were always measured simultaneously. Their slightly different noise patterns seem to indicate the noise of the device rather than that of the phantom. Figure 5(d) compares results for Lumogen and ICG from a bleaching experiment over 1 h. The data on Lumogen are constant apart from a small decrease (1%) within the first 10 min. The most likely reason is a small decrease of the LED emission power due to warm up which is not fully suppressed by alternating between 200-ms exposure and 600-ms recovery time.

The Lumogen phantoms did not show any change in the visible appearance over the 4 years, and new phantoms printed with the same composition have the same coloring as the previous ones. In particular, we did not observe any yellowing as known from phantoms made from epoxy resin or polyurethane. Moreover, storing of Lumogen cuboids under daylight for several months did not change their visible appearance. This was different for the cuboids with ICG exposed to daylight. They lost, in part, the dye-specific blue-green color compared to cuboids from the same badge stored in the dark.

Overall, the investigations on Lumogen phantoms indicate a genuine photostability, making such phantoms well-suited for performance monitoring, device comparison, and maintenance of hand imagers. In contrast, our stability investigations on phantoms printed with ICG do not confirm the high stability over time reported by Ruiz et al.^36^ These authors observed deviations between +4% and −4% (with 1% to 2% error bars) over 2 months without a systematic tendency. Possible reasons are the different base resin, the different printer types, and differences in the UV curing process. It is well known that ICG stability is very sensitive against environmental factors. Surprisingly, Ruiz et al.^35^^,^^36^ explained the stability of their phantoms by differences in the chemical structure between the applied “laser dye” (IR-125) and ICG, although the data sheet of the manufacturer (Exciton Inc., Lockburne, Ohio, United States) explicitly declares that IR-125 and ICG are two names for the same substance (CAS Registry Number: 3599–32–4).^46^ Our direct comparison of Lumogen and ICG phantoms illustrates that Lumogen behaves much more stable, which is in accordance with the known differences in stability between rylene and cyanine dyes.^47^

### 3.5 Homogeneity of Printed Phantoms

**Fig. 6:**
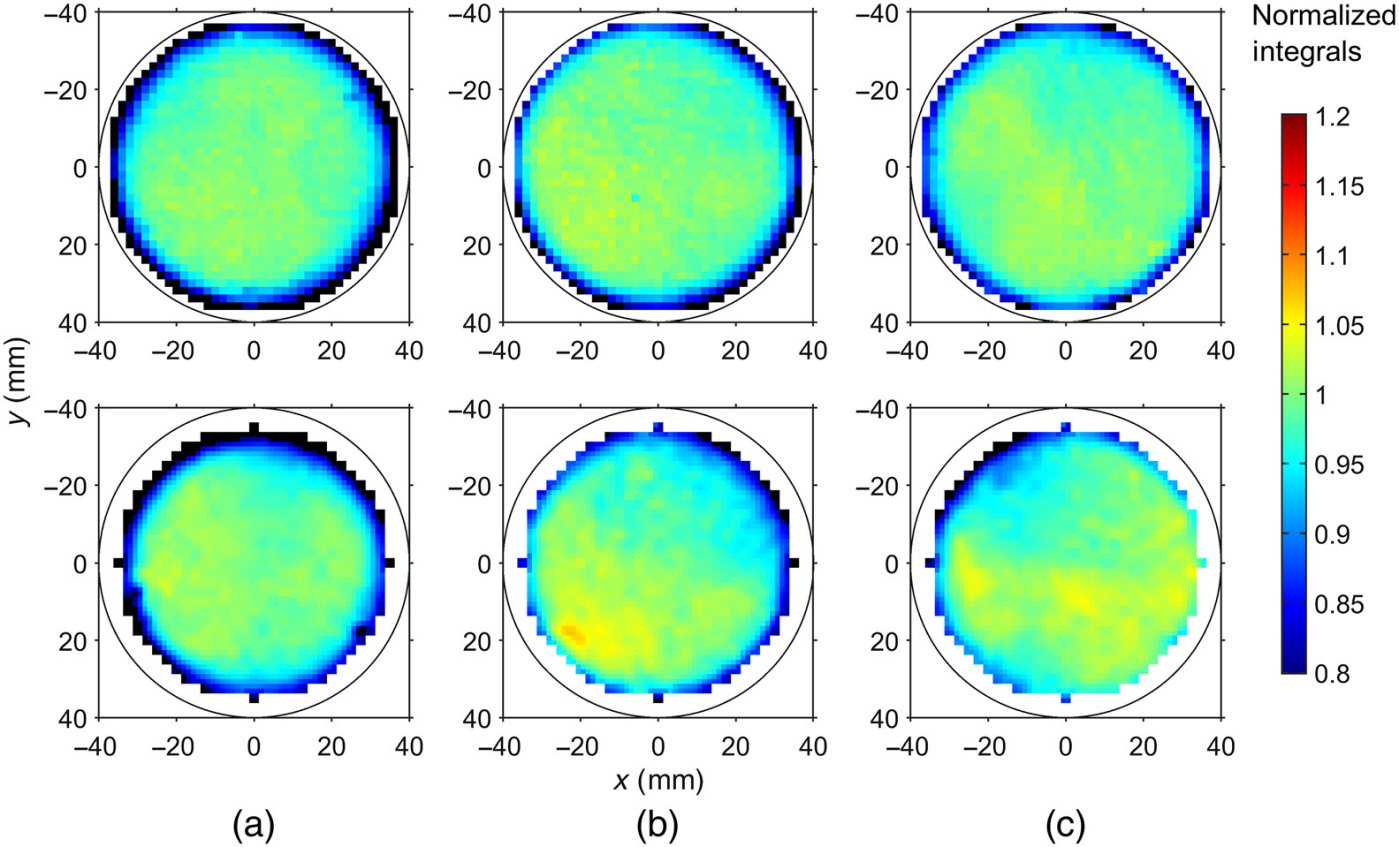
Normalized integrals of the DTOFs measured at the excitation wavelength (760 nm, top row) and at the fluorescence wavelength (bottom row) of the printed disc phantoms (2  mg/g ${\mathrm{TiO}}_{2}+0.5\;\mu L/g$ ink in E-Shell 600) with different Lumogen concentrations: (a) 0.5  μg/g Lumogen, (b) 1.5  μg/g Lumogen, and (c) 5  μg/g Lumogen. The circle indicates the physical boundary of the phantom.

Figure 6 illustrates the homogeneity of horizontally printed disc phantoms for three different concentrations of Lumogen. The images show normalized integrals of the measured DTOFs at 760 nm (excitation wavelength, measured with a 780-nm short-pass filter) and at the fluorescence wavelengths (measured with an 800-nm long-pass filter) over the scan position. Overall, the variations of the integrals in the central part of the images are smaller than 5%. There are two moderate deviations close to the phantom edges. The fluorescence image for 1.5  μg/g Lumogen [Fig. 6(b), bottom] shows a 7% increase in the lower left part and the fluorescence image for 5  μg/g Lumogen [Fig. 6(c), bottom] an 8% decrease in the upper left part. Overall, the homogeneity has an acceptable level. Further improvements may be obtained by continuously stirring the resin mixture in the bath of the printer. The decreasing values of the integrals close to the lateral edge of the phantoms are caused by photon losses through the sides of the phantom. These lossy regions are more extended at the fluorescence wavelength.

**Fig. 7:**
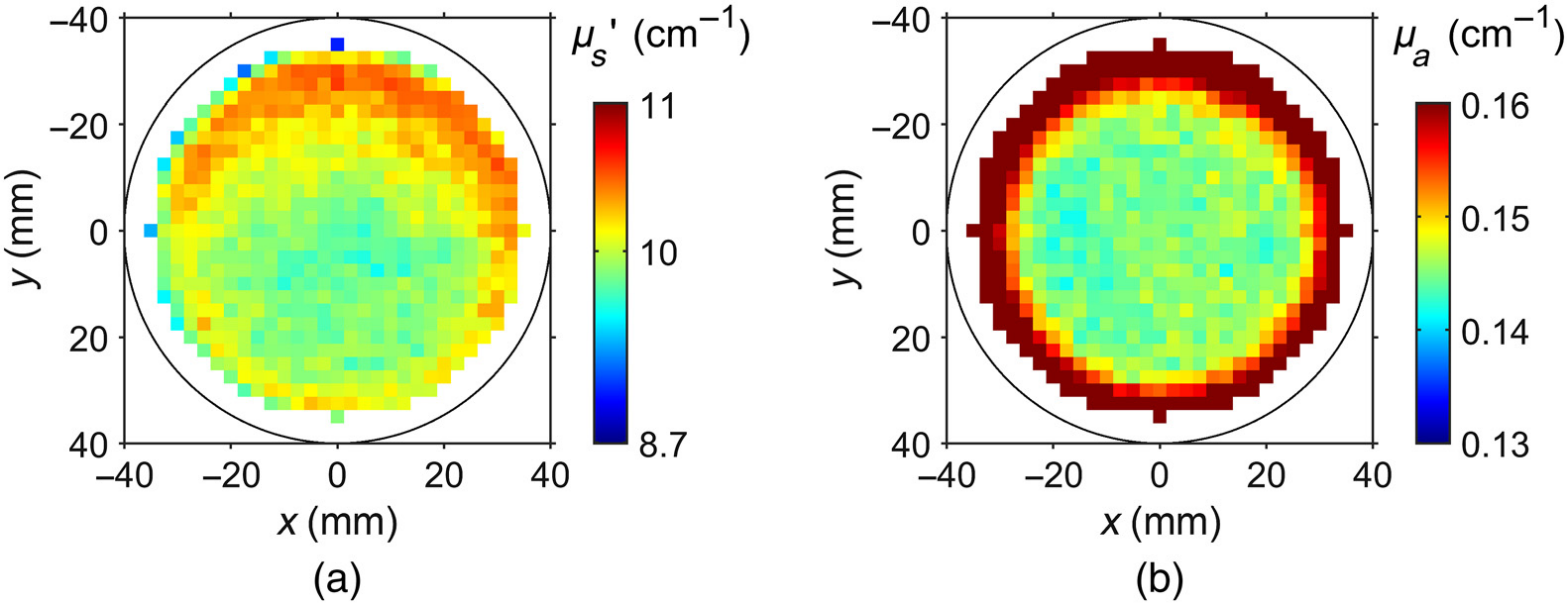
Example images of (a) reduced scattering and (b) absorption coefficients at $\lambda_{\mathrm{ex}}=760\;\mathrm{nm}$, derived by using the Monte Carlo model. Concentration: $2\;\mathrm{mg}/g\;{\mathrm{TiO}}_{2}+0.5\;\mu L/g$ ink in E-Shell 600 with 0.5  μg/g Lumogen.

In Fig. 7, an example is given for scattering and absorption coefficients derived at each scan position by application of the Monte Carlo model. Data correspond to the phantom with 0.5  μg/g Lumogen. Similar to Fig. 6, deviations in the central part are below ±5% for both properties indicating a good homogeneity. The absorption image shows much stronger edge effects than the corresponding integral data in Fig. 6(a). As mentioned above, these edge effects are due to photons leaving the phantom through the curved sides. Such photon losses are not taken into account by the geometry of the infinite slab used here. The increased reduced scattering coefficient along the upper phantom boundary could be caused, at least in part, by artificial structures on the phantom lateral surface indicating the fabrication number and the print direction. Besides, the slight increase of $\mu_{s}^{\prime }$ toward the phantom lateral edges is again an effect of the finite size of the disc compared to the infinitely extended slab in the model.

Compared with the horizontal printed phantoms, the vertical phantoms (not shown) show larger inhomogeneities in their optical properties caused by the fabrication process. Due to the geometry, the vertical printing orientation needs a longer 3D printing process, resulting in more inhomogeneous distributions of the ingredients in the base material. Continuous stirring of the resin bath during the fabrication process could prevent such effects, even though it is technically difficult to implement.

### 3.6 Phantoms with Application-Specific Shapes

**Fig. 8:**
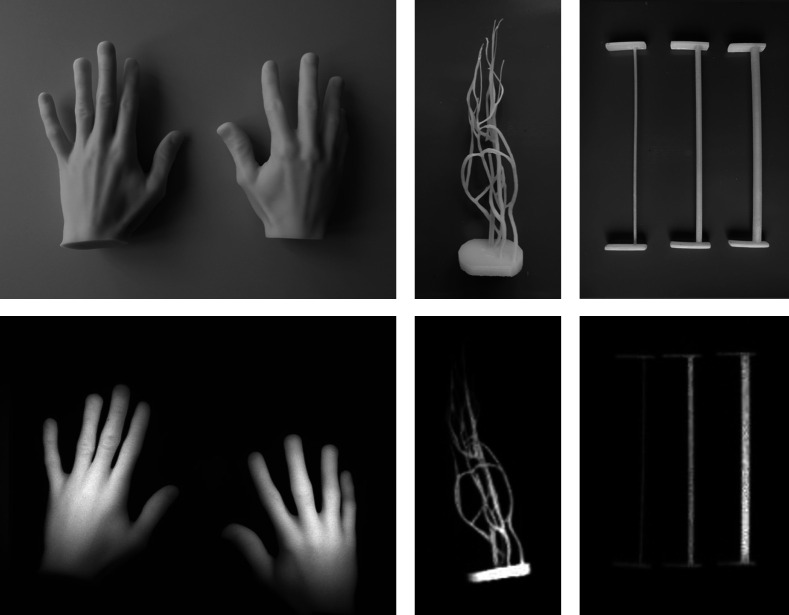
Photos (top row) and fluorescence images (bottom row) of printed phantoms. (a) Both hands of a volunteer, (b) vessel structure of a finger (with artificial base plate), (c) cylinders with 1-, 2-, and 3-mm diameter.

Figure 8 shows phantoms with shapes that are useful for studies with the hand imager. All these phantoms were printed with 1.5  μg Lumogen, 2 mg ${\mathrm{TiO}}_{2}$, and 0.5μL ink per gram of resin to mimic the conditions of *in vivo* investigations in the detection of rheumatoid diseases. The fluorescence counts for the hand models are in accordance with the intermediate data range during *in vivo* perfusion measurements with ICG. The higher brightness in the central parts of both hands is caused by the intensity profiles of the two LED groups being roughly centered on top of the hands. With hand phantoms of high homogeneity, the LED intensity profiles and the edge effects at the various boundaries could be studied in detail, and appropriate correction schemes could be developed to improve the devices. Besides hands of healthy persons, models of hands from patients with affected finger joints could be produced in a similar manner. The vessel structure of a finger in Fig. 8(b) contains vessels with diameters down to about 0.5 mm. The cylindrical structures [Fig. 8(c)] were originally printed with one base plate only. The second plate was printed separately and finally glued to the cylinder. These plates are helpful, e.g., in fixing the cylinders in finger-like molds to embed them in a scattering and absorbing environment. Similar, the vessel structure could be encapsulated by a nonfluorescent finger-like environment made, e.g., from a mixture of PDMS, ${\mathrm{TiO}}_{2}$, and ink.

## 4 Conclusion

We have demonstrated the feasibility of fabricating solid fluorescent phantoms with tissue-like optical properties in the NIR spectral range by a commercial 3D printer that uses UV curing of a methacrylate resin. By adding the fluorescent dye Lumogen IR 765 as well as ${\mathrm{TiO}}_{2}$ and ink as scattering and absorption ingredients to the base material it was possible to produce stable and durable phantoms with geometries and optical properties relevant for fluorescence imaging of rheumatoid diseases with ICG. The absorption maximum of Lumogen in the printed phantom is at about 760 nm, and fluorescence emission occurs at around 810 nm which fits well with the properties of ICG. Our long-term studies revealed an excellent photostability of the printed Lumogen phantoms over 4 years which was never reported so far for any other fluorescence phantom for NIR optical imaging of tissue. 3D printing of tissue-like phantoms with ICG dissolved in DMSO revealed a bad photostability which is in line with the instability issues generally known for ICG. Obviously, the recently reported high stability of 3D-printed phantoms with ICG^36^ over 2 months seems to be limited to particular printer types and resins. By printing Lumogen-based phantoms having the shape of the human hand and of a finger-like vessel structure we could demonstrate the fabrication of complicated structures relevant for the comparison of fluorescence hand imagers and for related performance checks.

Our results clearly represent progress in the customization of 3D printing photopolymer materials with user-defined fluorescence properties and long-term photostability. Our method is not limited to devices for fluorescence imaging of rheumatoid diseases. It has general potential to manufacture standardized tools for assessing the performance of optical diagnostic systems utilizing fluorescence of ICG, as well as for generating information on device working mechanism which may lead to improvements during clinical use.
